# An efficient copper-based magnetic nanocatalyst for the fixation of carbon dioxide at atmospheric pressure

**DOI:** 10.1038/s41598-018-19551-3

**Published:** 2018-01-30

**Authors:** Rakesh Kumar Sharma, Rashmi Gaur, Manavi Yadav, Anandarup Goswami, Radek Zbořil, Manoj B. Gawande

**Affiliations:** 1 Green Chemistry Network Centre, Department of Chemistry, University of Delhi, Delhi, 110007 India; 2 Regional Centre of Advanced Technologies and Materials, Department of Physical Chemistry, Faculty of Science, Palacký University Olomouc, Šlechtitelů 27, 783 71 Olomouc, Czech Republic; 3 Division of Chemistry, Department of Sciences and Humanities Vignan’s Foundation for Science, Technology and Research (VFSTR) Vadlamudi, Guntur, 522 213 Andhra Pradesh, India

## Abstract

In the last few decades, the emission of carbon dioxide (CO_2_) in the environment has caused havoc across the globe. One of the most promising strategies for fixation of CO_2_ is the cycloaddition reaction between epoxides and CO_2_ to produce cyclic carbonates. For the first time, we have fabricated copper-based magnetic nanocatalyst and have applied for the CO_2_ fixation. The prepared catalyst was thoroughly characterized using various techniques including XRD, FT-IR, TEM, FE-SEM, XPS, VSM, ICP-OES and elemental mapping. The reactions proceeded at atmospheric pressure, relatively lower temperature, short reaction time, solvent- less and organic halide free reaction conditions. Additionally, the ease of recovery through an external magnet, reusability of the catalyst and excellent yields of the obtained cyclic carbonates make the present protocol practical and sustainable.

## Introduction

In the present era of progressive global development, human activities, such as combustion of fossil fuels, deforestation and hydrogen production from hydrocarbons have contributed a lot towards raising the concentration of carbon dioxide (CO_2_) in the atmosphere^1^. Moreover, this rise is considered to be the major benefactor towards global warming and abnormal climate changes^2^. Hence, the mitigation of CO_2_ emission has become the serious *albeit* challenging issue for the countries, scientists and concerned area of research.

Consequently, a plethora of strategies have been developed for capturing and storage of CO_2_ (CCS)^3–6^. On the other hand, valorisation of CO_2_ into value-added compounds is considered as the enviable and attractive alternative to CCS^7^. This methodology is not only beneficial in controlling the CO_2_ concentration but also offers the feedstock for the synthesis of pharmaceutical compounds^8–10^. However, the consumption and utilization of CO_2_ on a large scale encounter major challenges due to its inherent thermal stability and kinetic inertness^11^. Moreover, CO_2_ has been recently recognised as environmentally benign, inexpensive, non-flammable, abundant and renewable C_1_ building block^12^.

One of the promising strategies for CO_2_ fixation is the cycloaddition reaction between epoxides and CO_2_ to obtain cyclic carbonates^13^. These cyclic carbonates are broadly utilized as electrolyte components in lithium batteries, green polar aprotic solvents, and intermediates for the production of plastics, pharmaceuticals and fine chemicals^14–17^. Several strategies including metal complexes of Cr, Co, Ni, Zn, Fe, N-heterocylic carbenes (NHCs), metal organic frameworks (MOFs) and ionic liquid-based protocols have been reported to facilitate the reaction of epoxides with CO_2_^18–30^. Despite significant benefits in terms of reactivity and selectivity of the catalytic systems, most of the catalysts possess one or more problems including high catalyst loading, long reaction time and tedious reaction procedure and catalyst preparation. Although, there are catalytic systems that facilitate the transformation of CO_2_ at atmospheric pressure and mild reaction conditions, yet there is always much scope available for the improvement at several levels^31–43^.

 During the past few years, heterogeneous catalyst systems offer a benign alternative to accomplish the organic transformations. Further advancement in the field of green chemistry and nanotechnology has introduced magnetically retrievable nanocatalysts which provide immense surface area, excellent activity, selectivity, recyclability and long lifetime^44–56^. Among various solid nanomaterials^57^, silica-coated magnetite nanosupports have garnered much attention, mainly because of their unique characteristics, such as chemical stability, non-toxicity, economic viability and simple preparation methods which can be practiced profitably by the industries. In addition to that, the fact that they are magnetically separable provides an alternative to cumbersome filtration and centrifugation techniques, saving time, energy as well as the catalyst^58,59^. As a part of our ongoing research work on advanced nanomaterials for the development of sustainability and nanocatalysis^58–65^, herein, we describe the synthesis and characterisation of an efficient, easily generated, copper-based magnetic nanocatalyst as Cu is abundant, inexpensive, less toxic, readily available and excellent catalyst in comparison to the earlier reported metals^66^. Notably, this catalytic system fixes the carbon dioxide under atmospheric pressure, solventless and organic halide free reaction conditions, rendering the present protocol sustainable, straightforward, superior and cost effective. To the best of our knowledge, this is the first report, wherein a copper-based magnetic nanocatalyst has been utilised for the direct conversion of CO_2_ and epoxides into cyclic carbonates under mild reaction conditions.

## Methods

### Materials and reagents

Tetraethyl orthosilicate (TEOS), 3-aminopropyltriethoxysilane (APTES) and 2-acetylbenzofuran (ABF) were purchased from Fluka, Alfa Aesar and Sigma Aldrich respectively. Ferrous sulphate heptahydrate and ferric sulphate hydrate were commercially obtained from Sisco Research Laboratory (SRL). All epoxides and other reagents were bought from Alfa Aesar and Spectrochem Pvt. Ltd. and used without further purification. Double distilled water was used for the synthesis and washing purposes.

### Characterisations

XRD peaks were recorded using a Bruker diffractometer (D8 discover) with 2θ range of 10–80° (scanning rate = 4°/min, λ = 0.15406 nm, 40 kV, 40 mA). TEM experiments were carried out on a FEITECHNAI (model number G^2^ T20) transmission electron microscope (operated at 200 kV). The elemental mappings were obtained by STEM-EDS with an acquisition time of 20 min. Sample preparation was performed by dispersion of powder samples in ethanol followed by ultrasonication for 5 min. One drop of this solution was placed on a copper grid with holey carbon film. The sample was dried at room temperature. Field emission-scanning electron microscopic analysis (FE-SEM) was carried out by a Tescan MIRA3 FE-SEM microscope. Powdered sample was immediately placed on metal stub covered with carbon tape. Sample was further sputter-coated with a JEOL JEC-3000 FC auto fine coater gold sputtering machine. The magnetisation of the samples was measured by VSM (Model number EV-9, micro sense, ADE). The FT-IR spectra were recorded using a PerkinElmer Spectrum 2000 and employing KBr disks. The amount of copper in the catalyst and filtrate was estimated by an inductively coupled plasma-optical emission spectrometer (ICP-OES) on Varian (Australia) Vista MPX equipped with an argon saturation assembly, CCD detector, and software 4.1.0 complying with 21 CFR 11. The products were analysed and verified by Agilent gas chromatography (6850 GC) with a HP-5MS 5% phenyl methyl siloxane capillary column (30.0 m × 0.25 mm × 0.25 µm) and quadrupole mass filter equipped 5975 mass selective detector (MSD) using helium as carrier gas.

The oxidation state of the copper in the catalyst was analysed using Omicron Make XPS system with monochromatised AlKα X-Ray radiation (1486.7 eV) with hemispherical energy analyser and resolution of 0.6 eV.

### Preparation of catalyst

**Figure 1:**
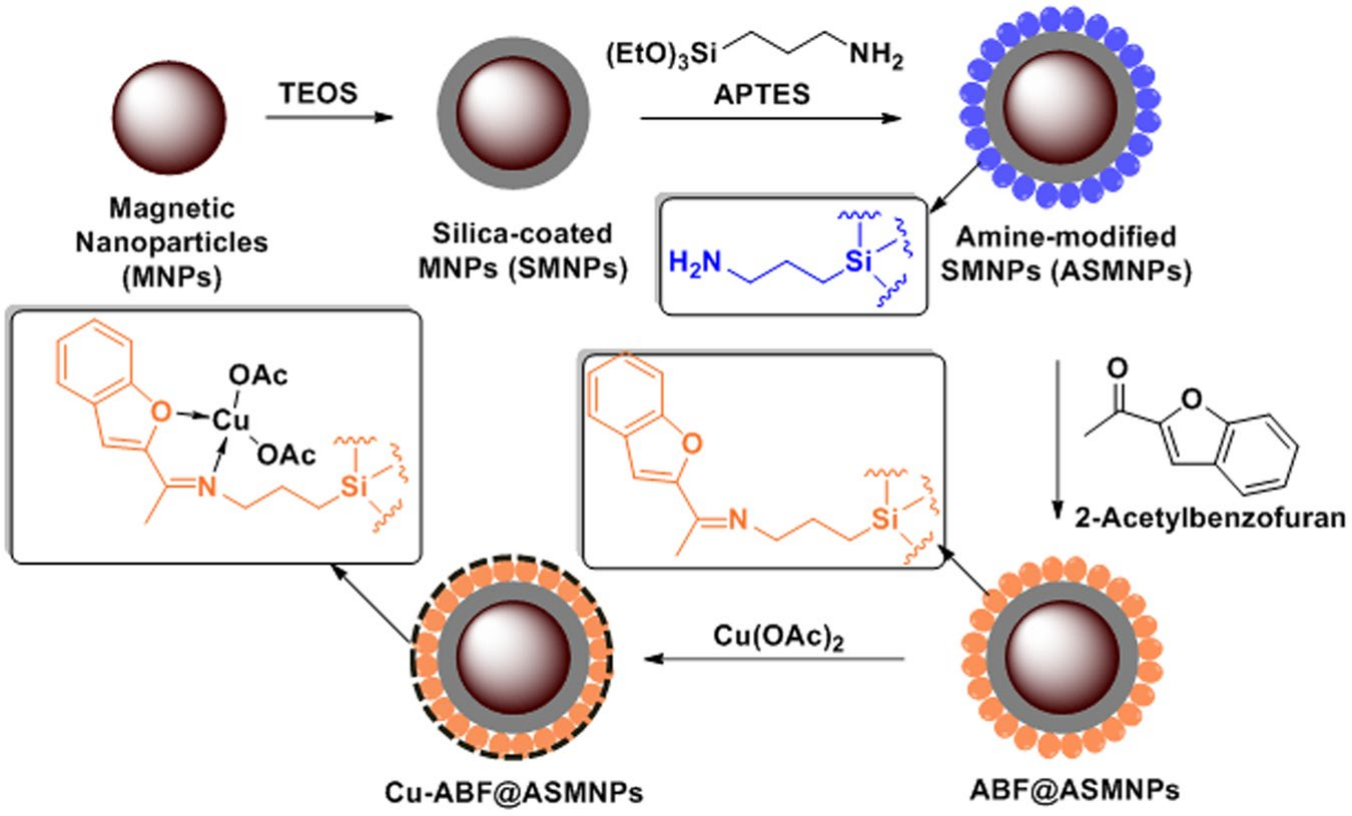
A schematic illustration of the formation of Cu-ABF@ASMNPs nanocatalysts.

The first step towards the synthesis of copper-based magnetic nanocatalysts involved the preparation of Fe_3_O_4_ magnetic nanoparticles (MNPs). In order to avoid agglomeration of MNPs, they were coated with silica, using TEOS to form silica-coated magnetic nanoparticles (SMNPs). For attaining the amine functionalised surface, we employed APTES to form amino-functionalised silica-coated magnetic nanoparticles ASMNPs. Further, the ligand 2-acetylbenzofuran (ABF) was immobilized on the surface of ASMNPs *via* Schiff base reaction to obtain ABF-grafted-ASMNPs (ABF@ASMNPs). Finally, the resultant nanoparticles were metalated with copper (II) acetate to obtain the final copper based magnetic nanocatalyst (Cu-ABF@ASMNPs) (Fig. 1).

### Synthesis of nanosupport composites

Magnetic (Fe_3_O_4_) nanoparticles were prepared by co-precipitation method^67^. Ferric sulfate hydrate (6.0 g) and ferrous sulfate heptahydrate (4.2 g) were dissolved in 250 mL water and stirred at 60 °C till yellowish-orange solution was obtained. Further, 25% NH_4_OH (15 mL) was added and the solution was stirred vigorously for 30 min. After some time, the colour of the bulk solution changed to black. The precipitated Fe_3_O_4_ nanoparticles were separated by an external magnet and washed several times with ethanol and water. Silica coating of these nanoparticles was carried out *via* a sol-gel technique^68^. A solution of 0.5 g Fe_3_O_4_ and 0.1 M HCl (2.2 mL) was prepared in the mixture of 200 mL ethanol and 50 mL water under sonication. Then 25% NH_4_OH (5 mL) was added to this solution followed by addition of 1 mL TEOS at room temperature. The mixture was subsequently stirred for 6 h at 60 °C. The obtained SMNPs were separated magnetically and washed with ethanol and water. Finally, 0.5 mL APTES was added to the solution of 0.1 g SMNPs in 100 mL ethanol and the resultant solution was stirred for 6 h at 50 °C. The derived ASMNPs were again separated magnetically and washed with water and ethanol and dried in vacuum oven.

### Preparation of Cu-ABF@ASMNPs catalyst

For the preparation of the catalyst, 4.0 mmol ABF and 2 g ASMNPs were added in 250 mL ethanol and refluxed for 3 h. The resultant 1 g ABF@ASMNPs was stirred with a solution of 2 mmol of copper acetate in 100 mL acetone for 4 h. Finally, the copper-based magnetic nanocatalyst was separated by an external magnet and dried in a vacuum oven.

### General reaction procedure for cycloaddition reaction of epoxide

In a dried 10 mL round bottom flask, 5 mmol of epoxide, 4 mol% of 1,8-Diazabicyclo(5.4.0)undec-7-ene (DBU) and 50 mg Cu-ABF@ASMNPs were added. The reaction mixture was stirred at 80 °C for 12 h under atmospheric pressure of CO_2_. After completion of the reaction, the catalyst was accumulated at the side of the vessel using an external magnet. Finally, the resulting solution was extracted with ethyl acetate and dried over anhydrous Na_2_SO_4_. The products were analysed and verified by GC-MS.

## Results and Discussion

### X-ray diffraction studies (XRD)

**Figure 2:**
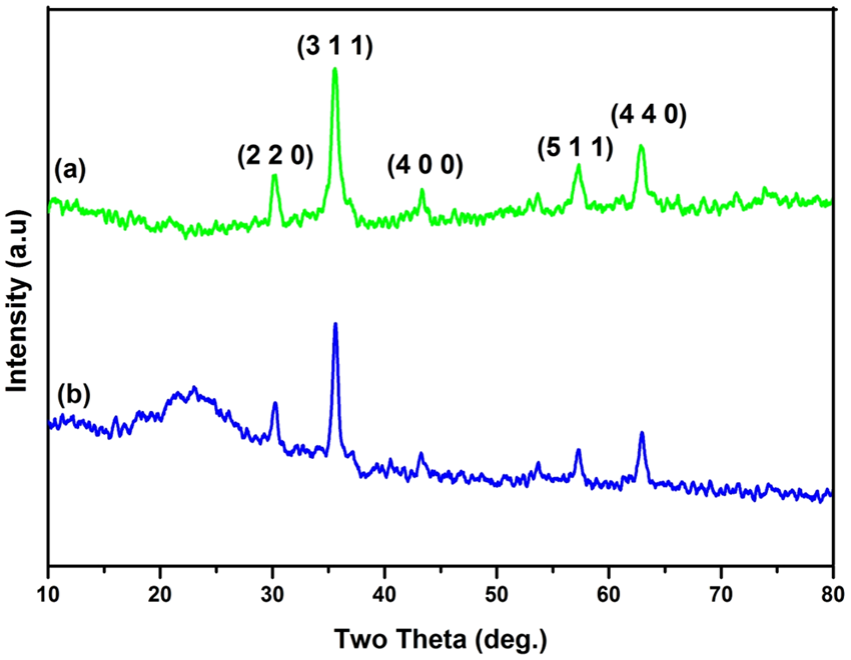
XRD pattern of (**a**) MNPs and (**b**) SMNPs.

The crystalline nature of the nanoparticles (MNPs and SMNPs), was confirmed by XRD studies. The diffraction peaks co-ordinated well with the standard XRD data of the Fe_3_O_4_ crystal with cubic inverse spinel structure when compared with the Joint Committee on Powder Diffraction Standards (JCPDS) database (card number, 19-0629) (Fig. 2a). The mean crystalline size of MNPs was measured by Debye–Scherrer equation and found to be 7.8 nm. After encapsulation with silica, a new broad peak at 23° appeared which indicates the presence of amorphous silica coating (JCPDS card number, 82-1574). All the other peaks of MNPs remained intact, exhibiting the phase stability of the magnetic nanoparticles (Fig. 2b)^69,70^. The XRD studies of catalyst Cu-ABF@ASMNPs were mentioned in Supplementary Fig. S1. Cu ions present in very low concentration in our Cu-ABF@ASMNPs. Therefore, signals for Cu nanoparticles (NPs) were not observed in the XRD pattern of Cu-ABF@ASMNPs.

### Fourier transform-infrared (FT-IR) spectroscopy

**Figure 3:**
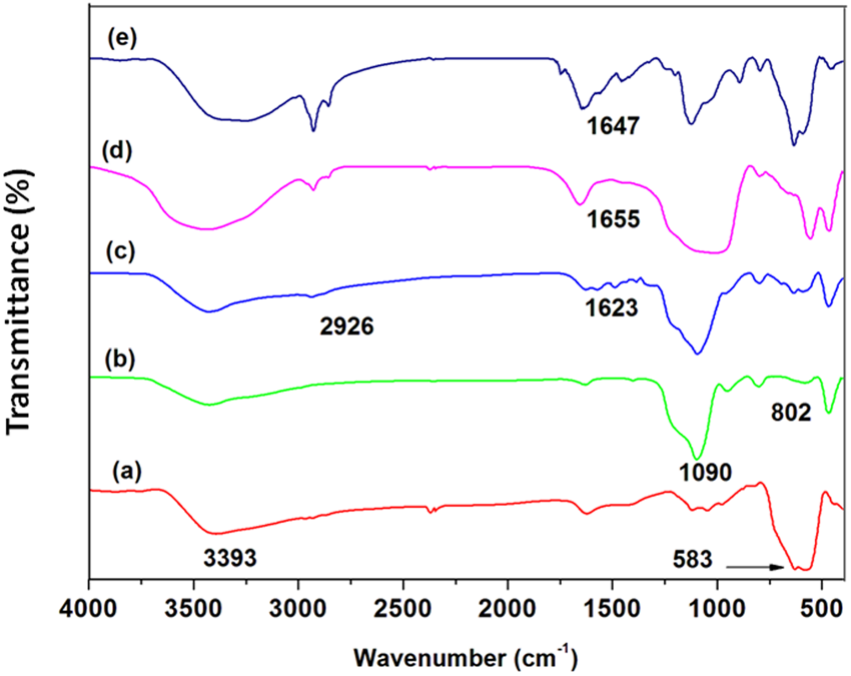
FT-IR spectra of (**a**) MNPs, (**b**) SMNPs, (**c**) ASMNPs, (**d**) ABF@ASMNPs and (**e**) Cu-ABF@ASMNPs.

The FT-IR spectroscopy was used to characterize the functionality of the resulting MNPs, SMNPs, ASMNPs, ABF@ASMNPs and Cu-ABF@ASMNPs (Fig. 3). FT-IR spectra revealed the distinctive peak at 583 cm^−1^ due to vibration of the Fe-O bond of the iron oxide nanoparticles (MNPs) and the broad peak at 3393 cm^−1^ of O-H stretching vibration due to absorbed water (Fig. 3a). The silica coating on the surface of MNPs was verified by peaks at 1090 cm^−1^ and 802 cm^−1^, ascribed to symmetrical and asymmetrical vibration of Si-O-Si bonds respectively (Fig. 3b). Functionalisation of aminopropyl group on the surface of SMNPs was confirmed by the emergence of new peaks at 1623 cm^−1^ and 2926 cm^−1^ which are assigned to the primary amine (-NH_2_) group and methylene (CH_2_) groups respectively (Fig. 3c). By comparing the spectra of ABF@ASMNPs (Fig. 3d) with those of ASMNPs, it is observed that after the Schiff condensation reaction the characteristic peak of imine group (C=N) appears at 1655 cm^–1^. On metalation, it is observed that the absorption at 1655 cm^−1^ shifted to 1647 cm^−1^ which confirms that copper is successfully anchored onto the surface of ABF@ASMNPs (Fig. 3e)^71–74^.

### Transmission electron microscopy (TEM) and field emission scanning electron microscopy analysis (FE-SEM)

**Figure 4:**
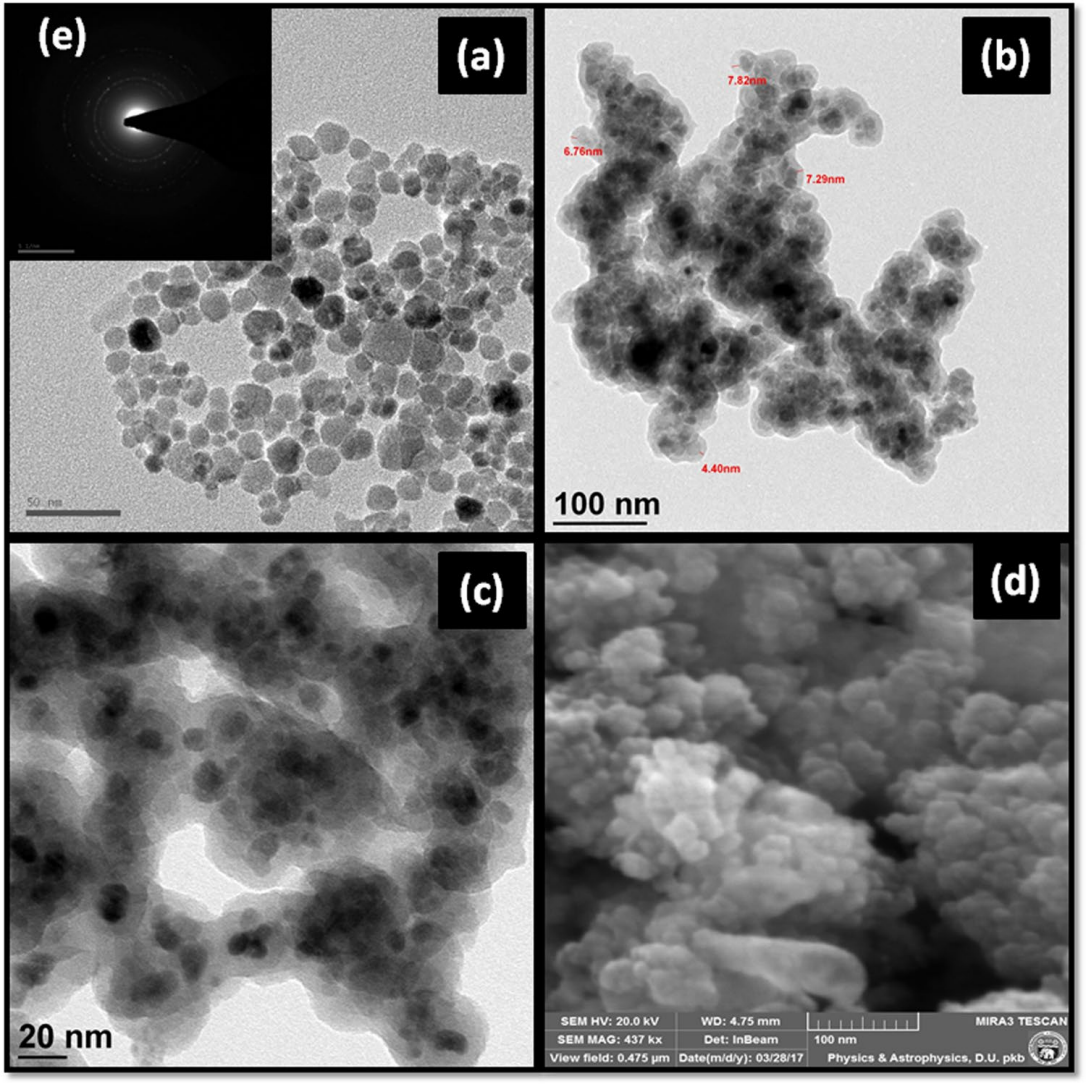
TEM images of (**a**) MNPs, (**b**) SMNPs, (**c**) Cu-ABF@ASMNPs catalyst, (**d**) FE-SEM image of Cu-ABF@ASMNPs and (**e**) SAED pattern of MNPs.

TEM micrograph of MNPs indicates that it is composed of tiny particles possessing the spherical morphology (Fig. 4a). Selected area electron diffraction (SAED) pattern of the MNPs is displayed as an inset in Fig. 4e. The white spots as well as the bright diffraction rings signify that the nanoparticles synthesised by the above-stated process are highly crystalline. The size distribution diagram of these nanoparticles shows that the average particle size of MNPs is in the range of 6–8 nm (see Supplementary Fig. S2). TEM image of SMNPs depicts the discrete core structure of MNPs of diameter of 6–8 nm encapsulated within the silica layer of 4–7 nm (Fig. 4b). Further, the TEM image of final Cu-ABF@ASMNPs nanocatalyst is demonstrated in Fig. 4c. The morphology of Cu-ABF@ASMNPs is characterized by FE-SEM as well. Fig. 4d clearly reveals that the catalyst is spherical in shape.

### Elemental mapping

**Figure 5:**
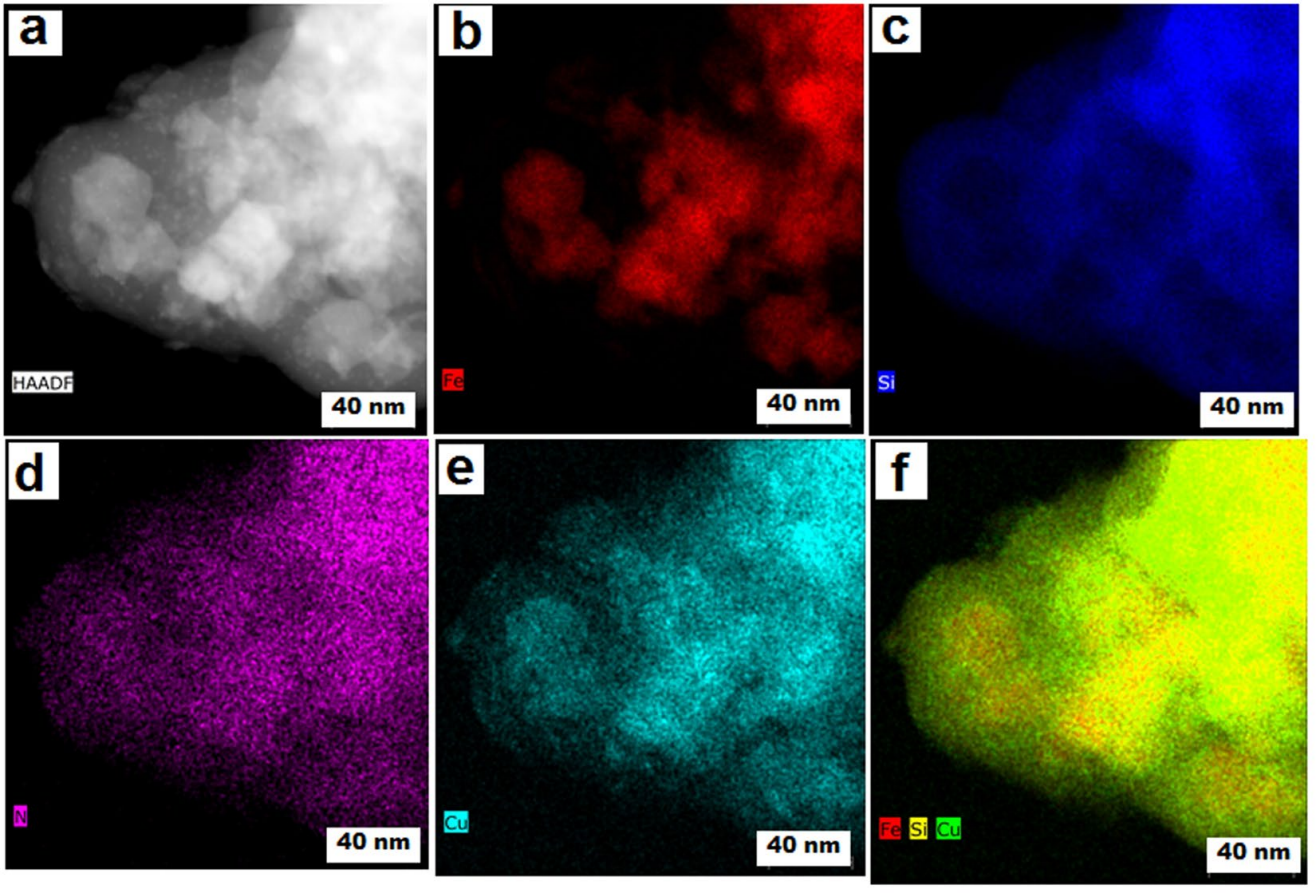
(**a**) HAADF image and (**b**–**f**) showing elemental mapping of Fe, Si, N, Cu and Fe/Si/Cu of Cu-ABF@ASMNPs sample.

Elemental mapping of the final Cu-ABF@ASMNPs catalyst is shown in Fig. 5a–f. The figures clearly reveal that Cu NPs dispersed uniformly on the surface of silica-based magnetite nano supports as mentioned in Fig. 1. The quantitative determination of the copper content was performed by the ICP-OES and was found to be 1.32 mmol/g in the final catalyst.

### X-ray photoelectron spectroscopy (XPS)

**Figure 6:**
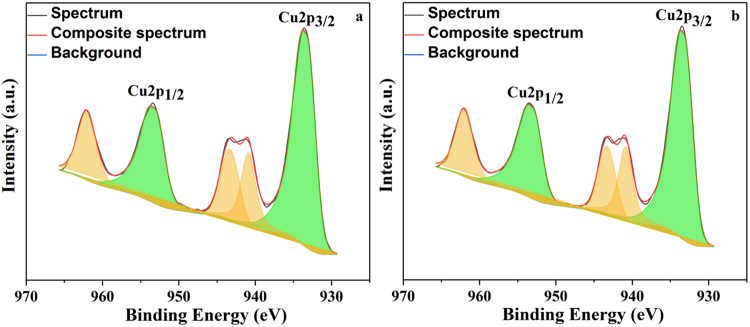
XPS Cu2p spectra of Cu-ABF@ASMNPs catalyst (**a**) fresh and (**b**) reused.

The chemical loading of the copper and its chemical state of copper in final catalyst as well as reused catalyst after 5 consecutive cycles was were measured by XPS technique, the spectrum is depicted in Fig. 6a,b. In both the samples the presence of Cu2p_3/2_ and Cu2p_1/2_ having binding energies 933.4 eV and 953.6 eV respectively along with the corresponding satellite bands having binding energies 940.9 eV, 943.4 eV and 962.1 eV confirm the presence of copper having 2+ oxidation state^75,76^. Further the survey spectra confirm the presence of silica, carbon and oxygen on the surface proves the coating of magnetic nanoparticles (see Supplementary Fig. S4).

### Vibrating sample magnetometer (VSM)

**Figure 7:**
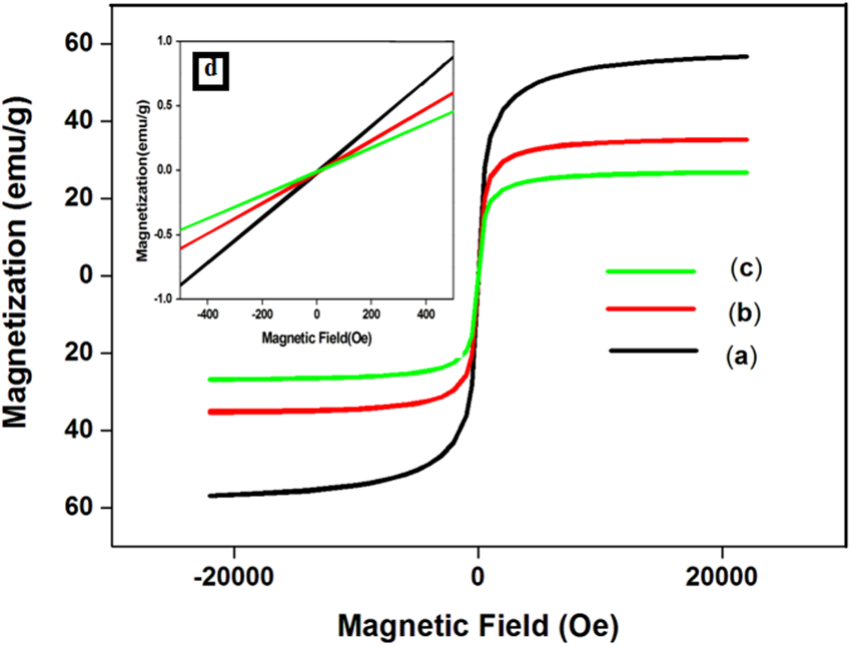
Magnetization curves obtained by VSM at room temperature for (**a**) MNPs, (**b**) SMNPs, (**c**) Cu-ABF@ASMNPs and (**d**) inset: enlarged image near the coercive field.

The magnetic properties of MNPs, SMNPs and Cu-ABF@ASMNPs catalyst were thoroughly studied using vibrating sample magnetometry at room temperature (Fig. 7). The curves show decrease in the saturation magnetization (M_s_) values from MNPs (58 emu/g) to SMNPs (36 emu/g) to the final Cu-ABF@ASMNPs nanocatalyst (26 emu/g). This decrease can be attributed to the presence of non-magnetic silica and other functionalised groups onto the surface of MNPs. Although the M_s_ values have decreased sequentially, the obtained nanocatalyst can be effortlessly removed from the reaction media *via* an external magnet. The curves also exhibit no hysteresis loop which indicates the superparamagnetic nature of these nanoparticles. Besides, this behaviour can be confirmed from the figure given in inset which shows negligible coercivity and remanence. This implies that as soon as the applied magnetic field is removed, the NPs would retain no residual magnetism, thereby making MNPs good candidates for catalytic support^77^.

### Catalytic activity of Cu-ABF@ASMNPs for cycloaddition reaction

**Figure 8:**
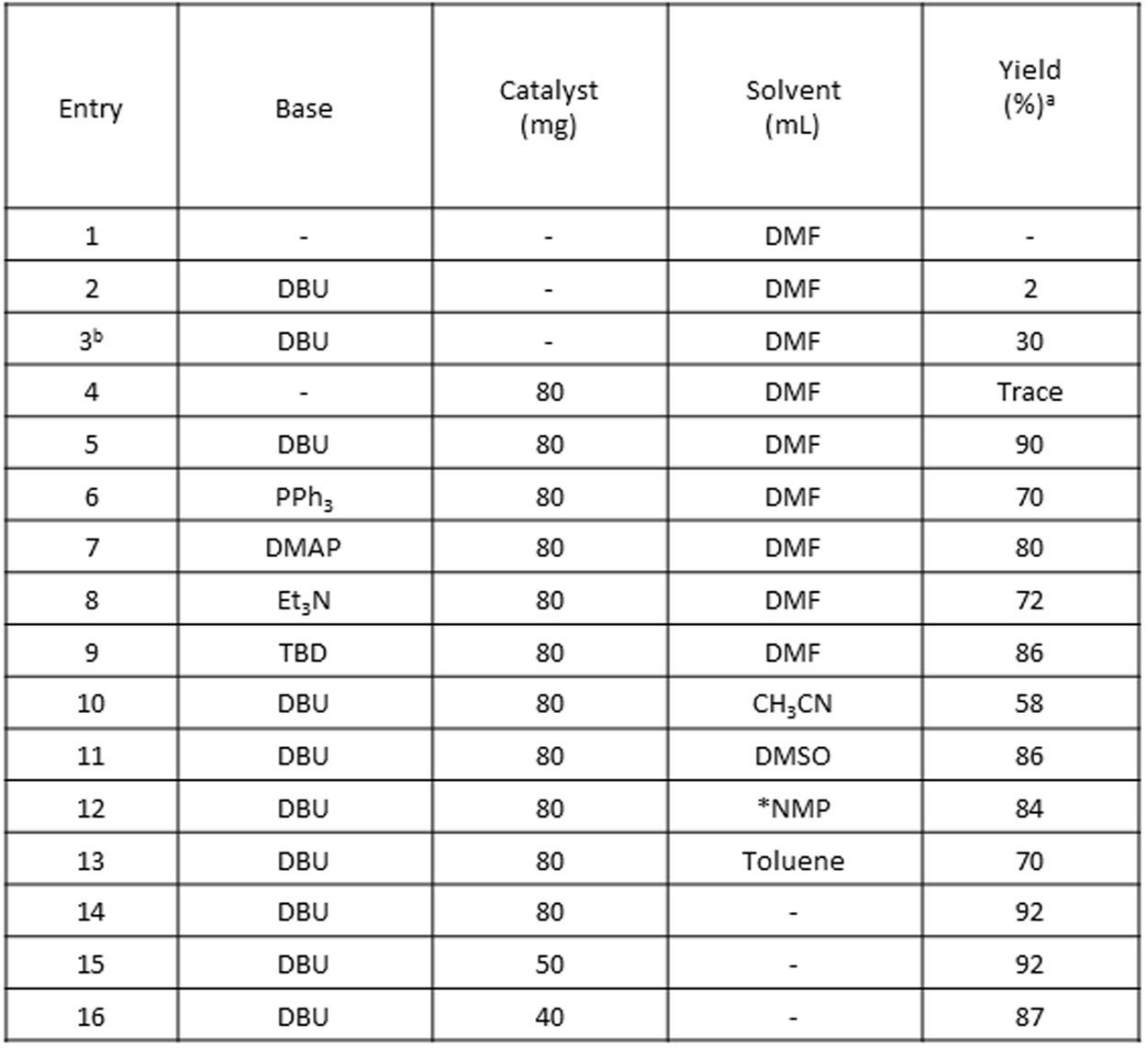
Cycloaddition reaction of styrene oxide with CO_2_. Reaction conditions: Styrene oxide (5 mmol), base (4 mol %), Cu-ABF@ASMNPs (40–80 mg), 12 h, 80 °C, under 1 atm of CO_2_. ^a^GC-MS yield. ^b^DBU (12 mol %) and 48 h. *NMP (N-Methyl-2-pyrrolidone).

Herein, the catalytic efficiency of copper-based magnetic nanocatalyst was investigated using styrene oxide as model substrate (Fig. 8). The screening of organic bases, solvents and amount of catalyst was carried out with optimised temperature and reaction time. Recently, organic bases have attracted widespread interest as CO_2_ activators which form the zwitterionic adduct, an activated form of CO_2_. We envisaged that assistance of these bases including DBU (1,8-Diazabicyclo(5.4.0)undec-7-ene), PPh_3_ (Triphenylphosphine), DMAP [4-(dimethylamino) pyridine], Et_3_N (Triethylamine) and TBD (1,5,7-triazabicyclo[4.4.0]dec-5-ene) could activate CO_2_ at atmospheric pressure^41,78–81^. As listed in Fig. 8, the reaction did not occur in absence of both the catalyst and base (entry 1). In presence of DBU only, 2% yield of styrene carbonate was observed when 4 mol% of DBU was used (entry 2) and with increase in amount of DBU to 12 mol%, improvement in yield was observed (entry 3). When the catalyst (Cu-ABF@ASMNPs) was used alone in absence of DBU, it was found to be almost inactive (entry 4) and this result proves the importance of DBU as CO_2_ activator. However, the combination of DBU and the catalyst afforded 90% yield of the desired product (entry 5) and this observation confirms the significance of our copper nanocatalyst.

Under the same reaction conditions, different organic bases were employed and DBU was found to be the best organic base than TBD, DMAP, Et_3_N and PPh_3_ for the activation of CO_2_ and provided the highest yield of styrene carbonate (entries 5–9). Next, we studied the effect of solvent in the cycloaddition reaction and we observed that among various solvents including DMF, DMSO, CH_3_CN, NMP and toluene, DMF appeared to be the best (entries 5, 10–13). When, the reaction was carried out under neat condition, to our surprise better yield of the product was found. Hence, it is confirmed that solvent does not play any significant role. Further, the catalytic amount was varied and 50 mg of the catalyst was found to be optimum to give the highest yield of styrene carbonate (entries 14–16).

**Figure 9:**
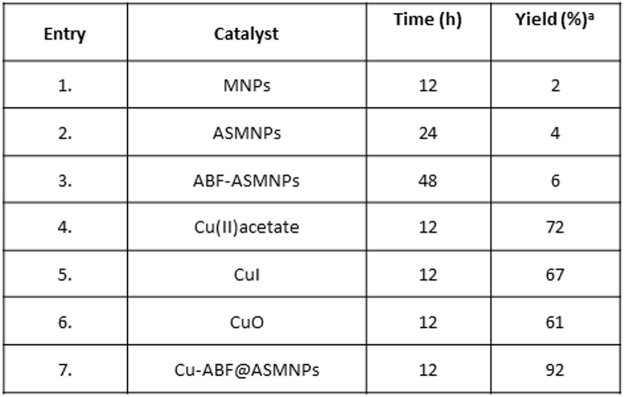
Screening of catalyst for cycloaddition reaction. Reaction conditions: Styrene oxide (5 mmol), DBU (4 mol %), catalyst (80 mg), 80 °C, under 1 atm of CO_2_. ^a^GC-MS yield.

**Figure 10:**
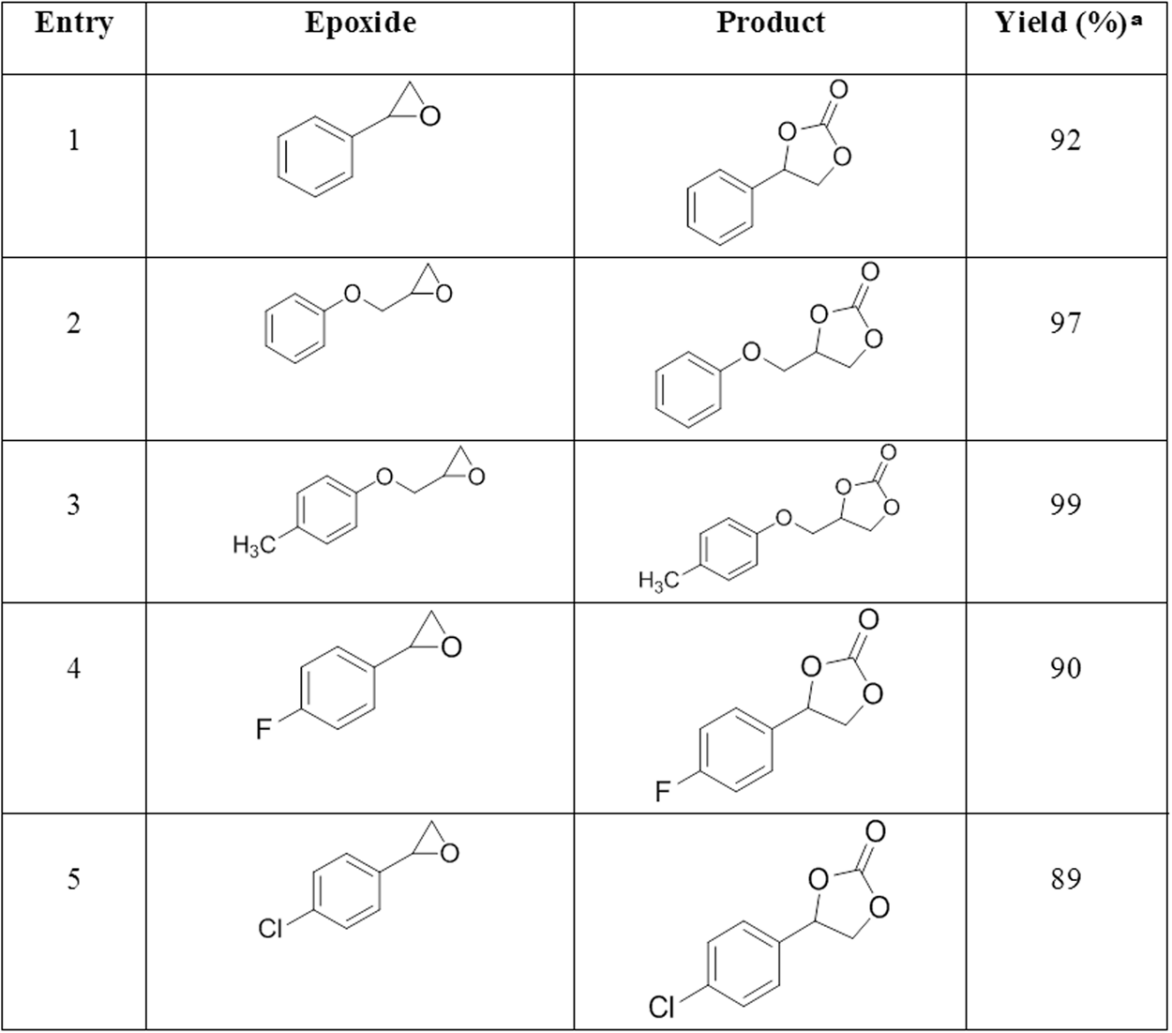
Conversion of various epoxides to cyclic carbonates under optimised condition. Reaction conditions: Epoxide (5 mmol), Cu-ABF@ASMNPs (50 mg), DBU (4 mol %), 1 atm CO_2_, 12 h, 80 °C. ^a^GC-MS yield.

When the precursor materials (namely ABF@ASMNPs, ASMNPs and MNPs) were used, no significant conversions of the styrene oxide were observed (Fig. 9, entries 1–3). The presence of copper-based source is extremely significant to catalyse the cycloaddition reaction of epoxide with CO_2_ (entries 4–6). The highest percentage was obtained in the case of Cu-ABF@ASMNPs, demonstrating the efficiency of the copper nanocatalyst in the cycloaddition reaction (entry 7). Under the optimised reaction conditions, this newly developed catalyst was then examined for other substrates as shown in Fig. 10. The results demonstrated that different epoxides converted to corresponding cyclic carbonates under mild conditions in high to excellent yields. The Cu-ABF@ASMNPs nanocatalyst also showed promising results in terms of mild reaction condition in comparison with the literature precedents (Table S1).

### Reusability of the catalyst

**Figure 11:**
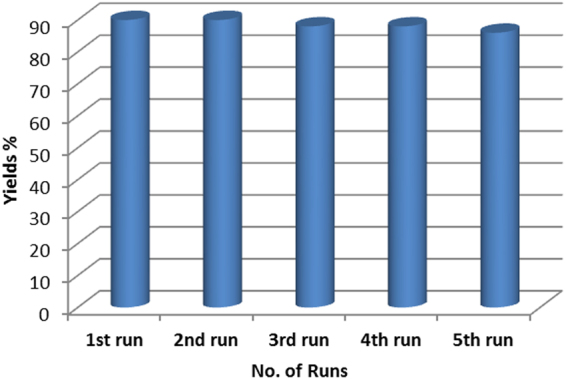
Catalytic recyclability test for successive five runs of styrene oxide. Reaction conditions: styrene oxide (5 mmol), DBU (4 mol %), Cu-ABF@ASMNPs (50 mg), 80 °C, 12 h, 1 atm of CO_2_.

Cu-ABF@ASMNPs nanocatalyst also be easily separated from the reaction mixture. To investigate the recyclability of the catalyst, styrene oxide was chosen as the substrate. After completion of the reaction, catalyst was separated from the reaction mixture using the external magnet, washed with ethyl acetate and ethanol and dried under vacuum. As shown in Fig. 11, catalyst could be recovered and reused at least five times, without any obvious decrease in catalytic activity.

Moreover, the TEM images of the fresh and recycled catalyst (after the fifth run) were very similar (see Supplementary Fig. S3). This means that the morphology of the catalyst remains unaltered after the reaction. Additionally, CHN analyses of fresh and recovered Cu-ABF@ASMNPs nanocatalyst were conducted and results are shown in Supplementary Table S2. The result of CHN analysis of recovered catalyst showed no significant change in C, H and N contents. These results indicate that heterogeneous Cu-ABF@ASMNPs catalyst has excellent recyclability for cycloaddition reaction.

### Hot filtration test

To test the heterogeneous nature of catalyst, a hot filtration test was carried out for cycloaddition reaction of styrene oxide under applied reaction condition. After completion of the reaction, catalyst was separated from the reaction mixture through external magnet and then filtrate was analysed by ICP-OES. After the ICP analysis, it was found that concentration of copper in the supernatant corresponds to negligible catalyst leaching (0.01 ppm). The supernatant was again transferred back into the reaction vessel and reaction was continued for further 6 h. No further conversion was observed after the separation of the catalyst.

An additional reaction was carried out at 80 °C for 2 h under the same reaction condition and after the catalyst was separated using an external magnet and the supernatant was again poured back into the reactor and the reaction was continued for additional 6 h and 12 h. It was observed that about 20% of conversion was obtained after 2 h and conversion of styrene oxide remained unchanged after 6 h. If this reaction continues for 12 h, 1% yield of styrene carbonate was obtained. This yield was due to DBU as mentioned in Fig. 8. It corroborated the view that the copper has not leached during the course of the reaction which further signifies the stability and heterogeneity of the prepared nanocatalyst.

### Mechanism

**Figure 12:**
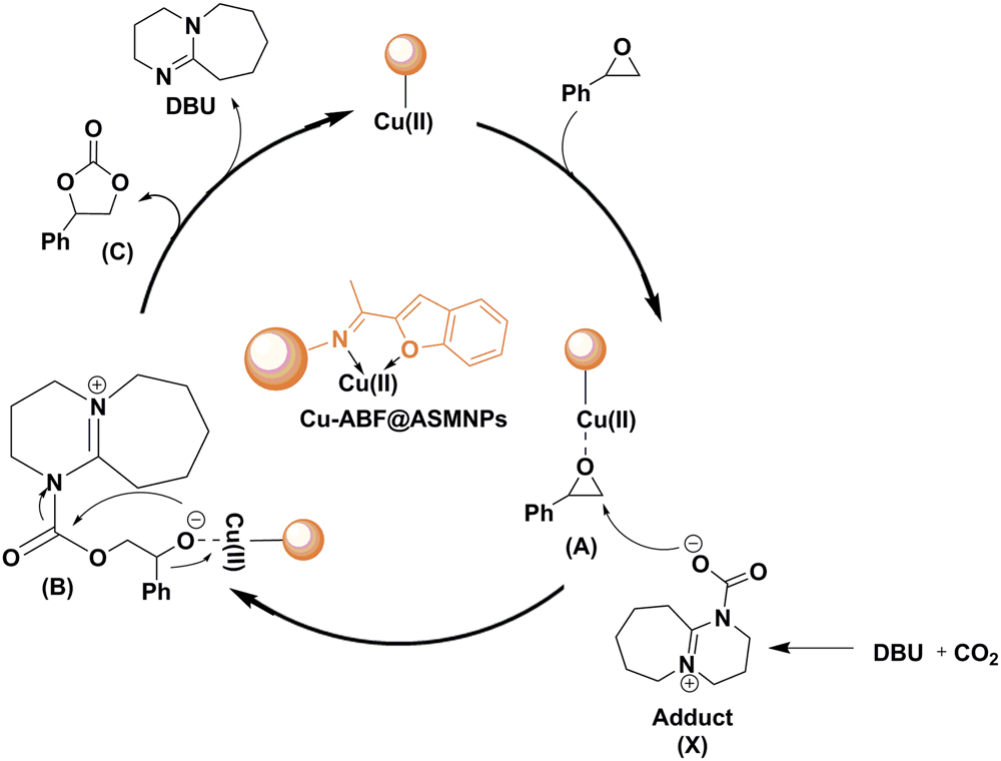
Plausible mechanism for the Cu-ABF@ASMNPs catalysed cycloaddition reaction of epoxide and CO_2_.

Firstly, Cu-ABF@ASMNPs catalyst activates the epoxide ring (**A**) which indicates the Lewis acidity of the catalyst plays a significant role in this organic transformation. Furthermore, DBU could activate CO_2_ *via* generation of zwitterionic adduct **X** (Fig. 12). The DBU-CO_2_ adduct may act as a nucleophile for the ring opening of epoxide. It attacks at less sterically hindered carbon atom of the epoxide which is activated by Cu-ABF@ASMNPs to generate the intermediate **B**. Then intramolecular cyclisation occurs to give cyclic carbonate (**C**) with regeneration of DBU and Cu-ABF@ASMNPs^41–43^. The activation of CO_2_ by DBU is essential to facilitate the ring-opening step and the combination of copper nanocatalyst/DBU system facilitates the cycloaddition reaction at mild reaction conditions.

## Conclusions

In summary, we have developed a copper-based magnetic nanocatalyst which has provided a straightforward way for the efficient fixation of CO_2_ through the cycloaddition reaction of epoxide and CO_2_ to form cyclic carbonates. The significant features of the present protocol include easy magnetic recovery, solvent-less and organic halide free reaction conditions. Moreover, the reactions proceeded under atmospheric pressure, and occurred at relatively short reaction time. Additionally, this simple methodology may serve as a benign alternative for conversion of CO_2_ and reusability of the catalyst up to five runs makes it favourable from the standpoint of environmental protection and resource utilization. This work might enlighten a promising strategy to construct efficient novel nanocatalyst for fixation of CO_2_ as renewable and environmentally friendly source of carbon in future.

## Electronic supplementary material

Supplementary Information
