# Supplementary material for: An efficient copper-based magnetic nanocatalyst for the fixation of carbon dioxide at atmospheric pressure
Source: Sci Rep. 2018 Jan 30;8:1901. doi: 10.1038/s41598-018-19551-3 (PMC5789884; doi:10.1038/s41598-018-19551-3)
Supplement: Supplementary file 1 — Supplementary Information [file 41598_2018_19551_MOESM1_ESM.doc]

**Supplementary Information**

An efficient copper-based magnetic nanocatalyst for the fixation of carbon dioxide at atmospheric pressure

Rakesh Kumar Sharma^1^,*, Rashmi Gaur^1^, Manavi Yadav,^1^ Anandarup Goswami^2^,

Radek Zboril^2^ and Manoj B. Gawande^2^,*

^1^Green Chemistry Network Centre, Department of Chemistry, University of Delhi, Delhi-110007, India. E-mail: rksharmagreenchem@hotmail.com (Rakesh Kumar Sharma)

^2^Regional Centre of Advanced Technologies and Materials, Department of Physical Chemistry, Faculty of Science, Palacký University Olomouc, Šlechtitelů 27, 783 71, Olomouc, Czech Republic. E-mail: manoj.gawande@upol.cz (Manoj B. Gawande)


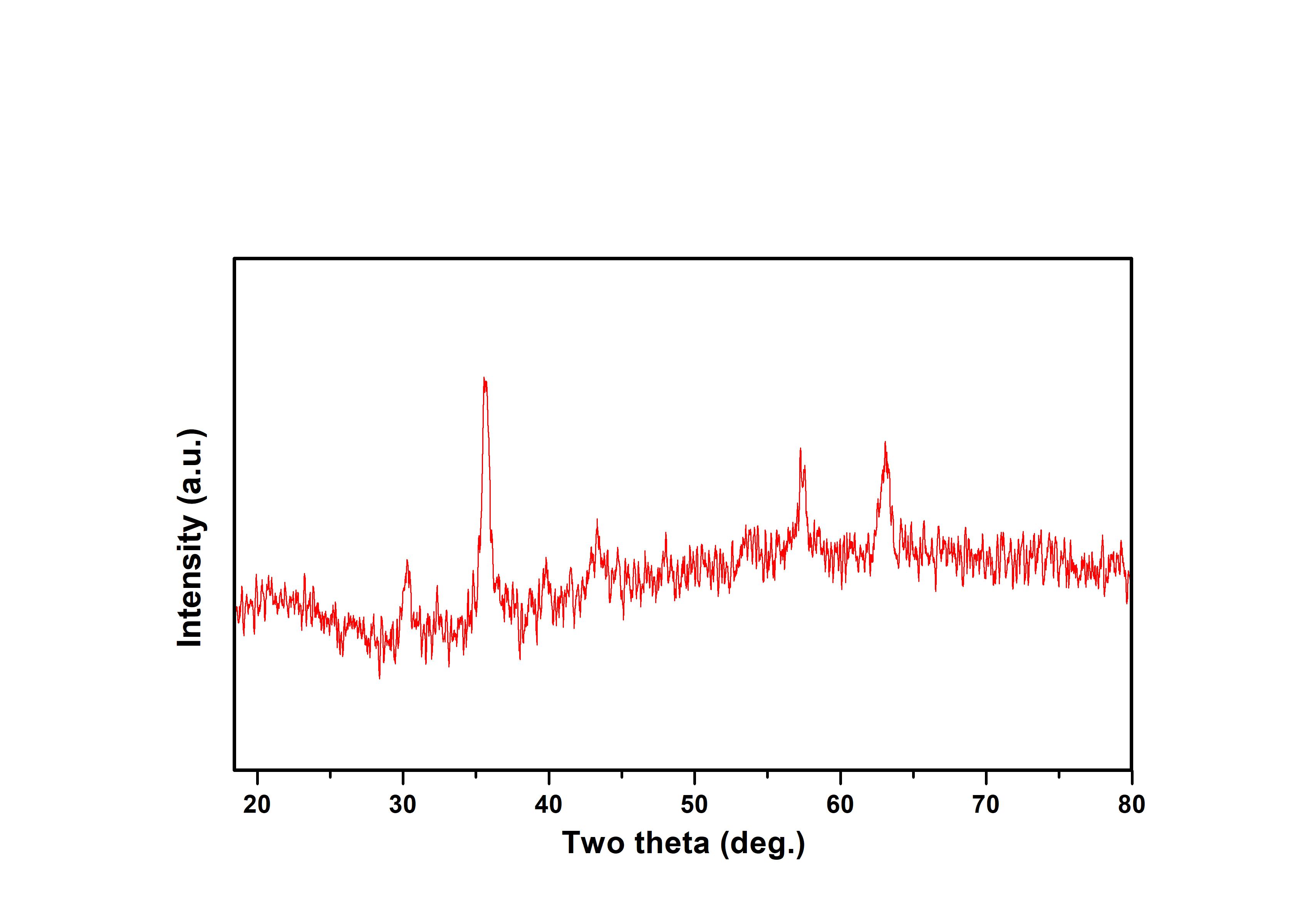


**Figure S1.** XRD pattern of Cu-ABF@ASMNPs.


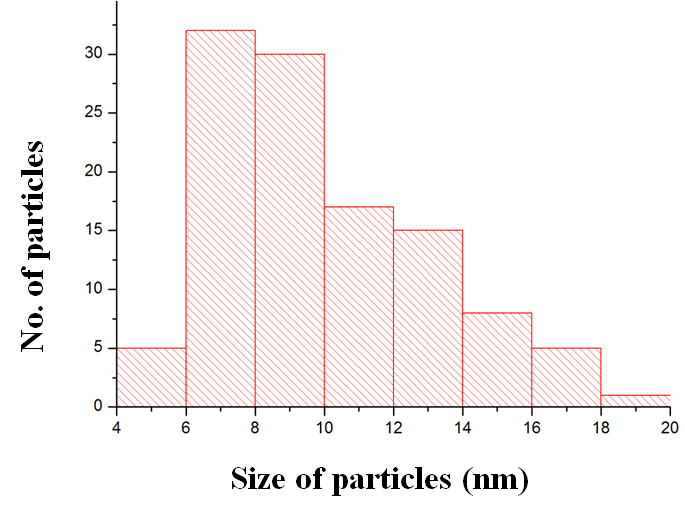


**Figure S2.** Size distribution curve of MNPs.


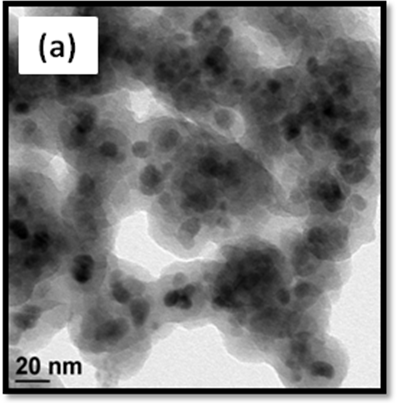

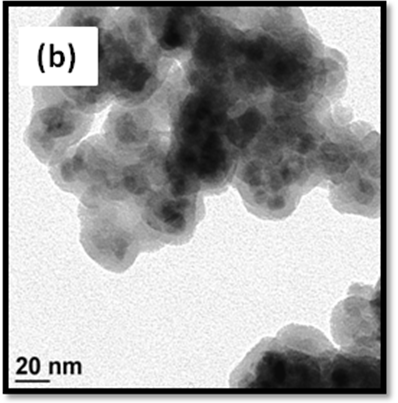


**Figure S3.** TEM images of the (a) fresh Cu-ABF@ASMNPs catalyst and (b) reused Cu-ABF@ASMNPs catalyst after five runs.


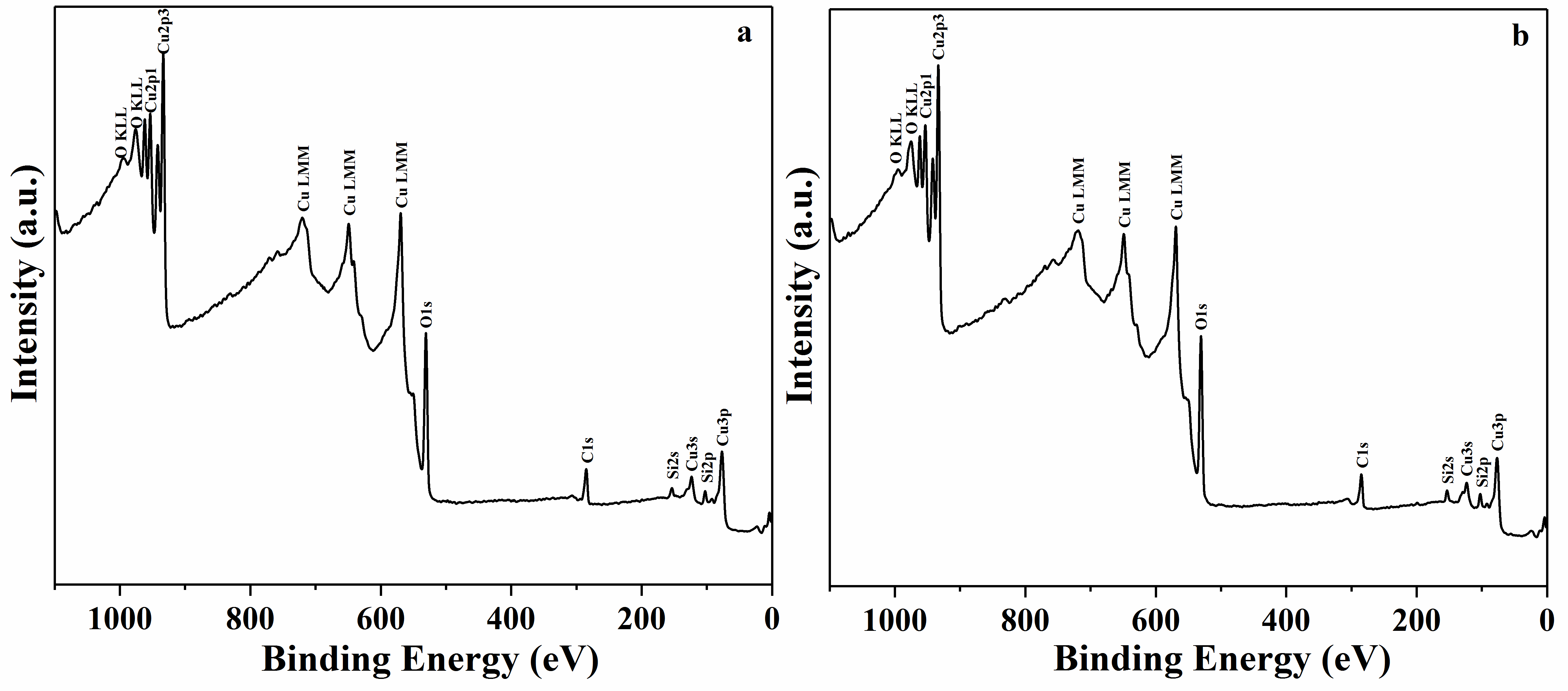


**Figure S4.** XPS survey spectra of fresh and reused Cu-ABF@ASMNPs catalyst.

**Table S1**. Comparison of the activity of the Cu-ABF@ASMNPs nanocatalyst with the previous catalytic system reported in the literature for the synthesis of cyclic carbonates.

| **No.** | **Epoxide** | **Catalyst** | **Reaction conditions** | **Yield (%)** | **Ref.** |
| --- | --- | --- | --- | --- | --- |
| 1 |  | BrTBDPEG_150_TBDBr  (Polyethyleneglycol functionalised basic ionic liquid) | 0.1 MPa CO_2_,  neat, 120 °C, 8 h | 93 | 1 |
| 2 |  | 2-pyridine methanol/nBu_4_NI | 1 atm CO_2_,  neat, 40 °C, 20 h | 85 | 2 |
| 3 |  | Hf-NU-1000 | 1 atm CO_2_,  nBu_4_NBr, rt, 56 h | 100 | 3 |
| 4 |  | Dinaphthyl silanediol | 1 atmCO_2_,  TBAI, rt, 18 h | 93 | 4 |
| 5 |  | Silica-supported pyrrolidinopyridinium iodide | 1 atm CO_2_,  neat, 100 °C, 20 h | 89 | 5 |
| 6 |  | Phosphorus ylide | 0.1 MPa CO_2_,  neat, 25 °C, 6 h | 62 | 6 |
| 7 |  | TETALiI | 1 atm CO_2_,  neat, 100 °C, 12 h | 90 | 7 |
| 8 |  | Zn(OH-salC_2_NH_2_Am) | 0.1 MPa CO_2_,  DMF, 120 °C, 12 h | 90 | 8 |
| 9 |  | Co/POP-TPP catalyst | 1atm CO_2_,  nBu_4_NBr, 29 °C, 48 h | 78 | 9 |
| 10 |  | 2,3-DhaTph COF | 1 atm CO_2_,  TBAI, 110 °C, 12 h | 94 | 10 |
| 11 |  | [Zn_6_(TATAB)_4_(DABCO)_3_  (H_2_O)_3_]·12DMF·9H_2_O | 1 atm CO_2_,  neat, 100 °C, 16 h | 89 | 11 |
| 12 |  | Bimetallic aluminium(salen) complexes | 1 atm CO_2_,  nBu_4_NBr, 25 °C, 24 h | 98 | 12 |
| 13 |  | Triazole-containing MOF | 1 atm CO_2_,  nBu_4_NBr, rt, 48 h | 96 | 13 |
| 14 |  | Amine-functionalised graphene oxide | 0.1 MPa CO_2_,  TBAI, 100 °C, 27 h | 94 | 14 |
| 15 |  | N-heterocyclic Carbenes | 0.1 MPa CO_2_,  neat, 120 °C, 24 h | 98 | 15 |
| 16 |  | Zr and Hf metal–organic frameworks | 1 atm CO_2_,  nBu_4_NBr, 25 °C, 60 h | 66 | 16 |
| **17** |  | **Cu-ABF@ASMNPs** | **1 atm CO_2_,**  **neat, 80 °C, 12 h** | **92** | **The present work** |

**Table S2**. Elemental analysis of the catalyst.

| No. | Sample | C [%] | H [%] | N [%] |
| --- | --- | --- | --- | --- |
| 1. | Fresh catalyst | 4.455 | 1.878 | 1.002 |
| 2. | Reused catalyst | 4.660 | 2.465 | 2.68 |

**References-**

1. Wang, L., Zhang, G., Kodama, K. & Hirose, T. An efficient metal- and solvent-free organocatalytic system for chemical fixation of CO_2_ into cyclic carbonates under mild conditions. *Green Chem.* **18,** 1229-1233 (2016).
2. Motokura, K., Itagaki, S., Iwasawa, Y., Miyaji, A. & Baba, T. Silica-supported aminopyridinium halides for catalytic transformations of epoxides to cyclic carbonates under atmospheric pressure of carbon dioxide. *Green Chem.* **11,** 1876-1880 (2009).
3. Beyzavi, M. H. *et al.* A hafnium-based metal–organic framework as an efficient and multifunctional catalyst for facile CO_2_ fixation and regioselective and enantioretentive epoxide activation. *J. Am. Chem. Soc.* **136,** 15861-15864 (2014).
4. Hardman‐Baldwin, A. M. & Mattson, A. E. Silanediol‐catalyzed carbon dioxide fixation. *ChemSusChem* **7,** 3275-3278 (2014).
5. Motokura, K., Itagaki, S., Iwasawa, Y., Miyaji, A. & Baba, T. Silica-supported aminopyridinium halides for catalytic transformations of epoxides to cyclic carbonates under atmospheric pressure of carbon dioxide. *Green Chem.* **11,** 1876-1880 (2009).
6. Zhou, H., Wang, G.-X., Zhang, W.-Z. & Lu, X.-B. CO_2_ adducts of phosphorus ylides: highly active organocatalysts for carbon dioxide transformation. *ACS Catal.* **5,** 6773-6779 (2015).
7. Luo, X., Chen, K., Li, H. & Wang, C. The capture and simultaneous fixation of CO_2_ in the simulation of fuel gas by bifunctionalized ionic liquids. *Int. J. Hydrogen Energy* **41,** 9175-9182 (2016).
8. Lang, X.-D., Yu, Y.-C. & He, L.-N. Zn-salen complexes with multiple hydrogen bonding donor and protic ammonium bromide: bifunctional catalysts for CO_2_ fixation with epoxides at atmospheric pressure. *J. Mol. Catal. A: Chem.* **420,** 208-215 (2016).
9. Dai, Z. et al. Metalated porous porphyrin polymers as efficient heterogeneous catalysts for cycloaddition of epoxides with CO2 under ambient conditions. J. Catal. 338, 202-209 (2016).
10. Saptal, V., Shinde, D. B., Banerjee, R. & Bhanage, B. M. State-of-the-art catechol porphyrin COF catalyst for chemical fixation of carbon dioxide via cyclic carbonates and oxazolidinones. *Catal. Sci. Technol.* **6,** 6152-6158 (2016).
11. Han, Y.-H., Zhou, Z.-Y., Tian, C.-B. & Du, S.-W. A dual-walled cage MOF as an efficient heterogeneous catalyst for the conversion of CO_2_ under mild and co-catalyst free conditions. *Green Chem.* **18,** 4086-4091 (2016).
12. Clegg, W., Harrington, R. W., North, M. & Pasquale, R. Cyclic carbonate synthesis catalyzed by bimetallic aluminium–salen complexes. *Chem. Eur. J.* **16,** 6828-6843 (2010).
13. Li, P.-Z. *et al.* A triazole-containing metal–organic framework as a highly effective and substrate size-dependent catalyst for CO_2_ conversion. *J. Am. Chem. Soc.* **138,** 2142-2145 (2016).
14. Saptal, V. B., Sasaki, T., Harada, K., Nishio‐Hamane, D. & Bhanage, B. M. Hybrid amine‐functionalized graphene oxide as a robust bifunctional catalyst for atmospheric pressure fixation of carbon dioxide using cyclic carbonates. *ChemSusChem* **9,** 644-650 (2016).
15. Talapaneni, S. N. *et al.* Nanoporous polymers incorporating sterically confined N-heterocyclic carbenes for simultaneous CO_2_ capture and conversion at ambient pressure. *Chem. Mater.* **27,** 6818-6826 (2015).
16. Zheng, J., Wu, M., Jiang, F., Su, W. & Hong, M. Stable porphyrin Zr and Hf metal–organic frameworks featuring 2.5 nm cages: high surface areas, SCSC transformations and catalyses. *Chem. Sci.* **6,** 3466-3470 (2015).
